# A randomized, double-blind, placebo-controlled, multiple-dose, parallel-group clinical trial to assess the effects of teduglutide on gastric emptying of liquids in healthy subjects

**DOI:** 10.1186/1471-230X-14-25

**Published:** 2014-02-12

**Authors:** Jolene Kay Berg, Eric H Kim, Benjamin Li, Bo Joelsson, Nader N Youssef

**Affiliations:** 1 DaVita Clinical Research, 825 S. 8th Street, Suite 300, Minneapolis, MN 55404, USA; 2 NPS Pharmaceuticals, Inc, 550 Hills Drive, Bedminster, NJ 07921, USA

**Keywords:** Teduglutide, Gastric emptying, Pharmacokinetics, Pharmacodynamics

## Abstract

**Background:**

Teduglutide, a recombinant analog of human glucagon-like peptide (GLP)-2, is a novel therapy recently approved for the treatment of adult patients with short bowel syndrome who are dependent on parenteral support. Previous studies assessing the effect of GLP-2 on gastric emptying in humans have yielded inconsistent results, with some studies showing no effect and others documenting a GLP-2–dependent delay in gastric emptying. The primary objective of this study was to assess the effect of teduglutide on gastric emptying of liquids in healthy subjects, as measured by the pharmacokinetics of acetaminophen.

**Methods:**

This double-blind, parallel-group, single-center study enrolled and randomized 36 healthy subjects (22 men, 14 women) to receive subcutaneous doses of teduglutide 4 mg or placebo (2:1 ratio; 23:13) once daily on Days 1 through 10 in the morning. Gastric emptying of a mixed nutrient liquid meal was assessed by measuring acetaminophen levels predose and at 0.25, 0.5, 0.75, 1, 1.25, 1.5, 2, 3, 3.5, 4, 5, 6, 8, 10, 12, and 14 hours after administration of 1000 mg acetaminophen on Days 0 and 10. The primary study endpoint was a pharmacokinetic analysis of acetaminophen absorption in subjects receiving teduglutide or placebo.

**Results:**

No significant differences in gastric emptying of liquids (acetaminophen area under the concentration [AUC] vs time curve from time 0 to the last measurable concentration, AUC extrapolated to infinity, maximum concentration [C_max_], and time to C_max_) were observed on Day 10 in subjects receiving teduglutide 4 mg versus subjects receiving placebo. There were no serious adverse events (AEs), deaths, or discontinuations due to an AE reported during the study.

**Conclusions:**

Teduglutide 4 mg/day for 10 days does not affect gastric emptying of liquids in healthy subjects as measured by acetaminophen pharmacokinetics. No unexpected safety signals were observed.

**Trial registration:**

This study was registered at ClinicalTrials.gov, identifier NCT01209351.

## Background

The proglucagon gene yields a single mRNA transcript expressed in the intestines, pancreas, and central nervous system (CNS) [1-3]. Tissue-specific cleavage of the resulting proglucagon precursor protein generates several peptides with distinct biological activity [3]. In the intestines, the proglucagon-derived proteins glucagon-like peptide (GLP)-1 and GLP-2 are expressed in the enteroendocrine L cells and are secreted in response to food intake [4-6]. The primary effects of GLP-1 are to increase postprandial insulin levels, inhibit glucagon secretion, and slow gastric emptying [7-15], whereas the primary effects of GLP-2 are to increase growth of intestinal epithelium, maintain intestinal mucosal morphology and function, and regulate energy intake [16-19]. Unlike GLP-1, GLP-2 has limited effect on insulin secretion or glucose homeostasis [20-22]. Furthermore, in contrast to GLP-1, GLP-2 does not decrease pancreatic glucagon secretion [21,23].

Multiple animal and human studies have demonstrated a delay in gastric emptying with GLP-1 or glucagon, another protein encoded by the proglucagon gene [7-12,24,25]. Because GLP-1 and GLP-2 are both derived from proglucagon and secreted following nutrient stimulation, it has been hypothesized that they coregulate gastric motility [26]. Indeed, in preclinical studies, GLP-2 significantly decreased the amplitude and frequency of postprandial gastric contractions and promoted gastric muscle relaxation [26,27]. However, conflicting results have emerged from studies on human subjects, with some reporting no effect of GLP-2 and others demonstrating a GLP-2–dependent inhibition of gastric emptying [21,28-30]. The divergent outcomes may have been influenced by differences between the studies, including variations in the amounts of GLP-2 delivered, the methods used to evaluate gastric emptying, and the content of test meals administered [21,28-30].

Teduglutide, a recombinant GLP-2 analog, is a novel therapy recently approved for the treatment of adult patients with short bowel syndrome (SBS) who are dependent on parenteral support [31,32]. SBS is defined as a clinically significant reduction in intestinal absorptive capacity resulting from surgical resection of the intestine due to disease, trauma, congenital defects, or complications of surgery [33,34]. Teduglutide promotes expansion of the remaining normal intestinal epithelium by increasing villus height and crypt depth in the small bowel mucosa, leading to increased absorptive area [31,32,35]. In a phase III, placebo-controlled trial, teduglutide significantly reduced parenteral nutrition and/or intravenous fluid (PN/IV) volume requirements and the number of infusion days required in patients with SBS [36].

Teduglutide is a synthetic protein that differs from native GLP-2 by the substitution of glycine for alanine at the second position from the N-terminus [37]. This single amino acid substitution renders it resistant to degradation by dipeptidyl peptidase-4 [37,38]. As a result, the half-life (t_½_) of teduglutide is increased compared with native GLP-2 following subcutaneous injection (t_½_ of 180–330 minutes vs 60–90 minutes, respectively) [39,40]. Because GLP-2 reduces gastric motility and delays gastric emptying, at least in animal and some human studies [26,27,29,30], it is possible that teduglutide, a similar but distinct molecular entity, may also inhibit gastric emptying. Modification of gastric emptying rate may alter the bioavailability of concomitantly administered drugs and modulate drug-drug interactions [41]. Thus, given the recent approval of teduglutide, clarification of the physiological effects of this novel peptide would provide important information to the medical community.

The primary objective of this study was to assess the effect of teduglutide on gastric emptying of liquids in healthy subjects as gauged by acetaminophen pharmacokinetics (PK), an accepted measure of gastric emptying kinetics [42].

## Methods

### Study design

**Figure 1:**
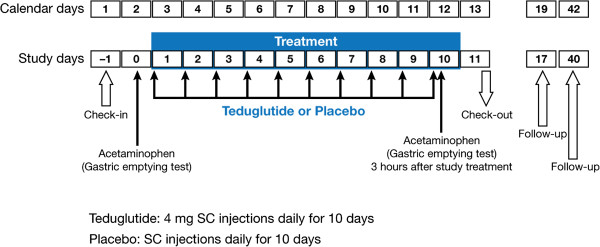
**Study design.** SC = subcutaneous.

In this double-blind, single-center, US-based, parallel-group study, 36 healthy domiciled subjects were randomized in a 2:1 ratio to receive subcutaneous teduglutide 4 mg or placebo daily for 10 days (Figure 1).

Subjects were randomized according to a randomization scheme generated by the sponsor or their designee. All doses of study drug for each subject were taken from the kit designated for that subject. Both subjects and investigators were blinded to the identity of the study drug administered; vials containing teduglutide and placebo were identical in appearance.

The RCRC Independent Review Board, LLC, of Austin, TX approved the study protocol and informed consent procedure before study initiation. The study was conducted in accordance with the Declaration of Helsinki, applicable International Conference on Harmonisation Guidelines, and Good Clinical Practices. This study was registered at ClinicalTrials.gov, identifier NCT01209351.

### Subjects

The study enrolled adults aged 18 to 45 years in good health who provided informed consent for participation. The study was conducted in healthy volunteers to eliminate the confounding effects of gastrointestinal (GI) disease. Key exclusion criteria included history of GI abnormality that could affect GI motility (including small bowel or colonic resection, inflammatory bowel disease, irritable bowel disease, and colon or GI tract cancer), allergy or sensitivity to acetaminophen, history of hepatitis or pancreatitis, evidence of liver inflammation, pregnancy or lactation, and body mass index >30 kg/m^2^. Prohibited prior and concomitant medications were generally those that may have affected gastric emptying, confounded efficacy or safety measurements, potentially posed a safety concern, or adversely potentiated or antagonized study drug treatment. Subjects were asked to abstain from alcoholic beverages and/or other alcohol-containing products from 48 hours before check-in until the last scheduled evaluation and blood sample collection before discharge on Day 11. Caffeinated beverages were not allowed during the gastric emptying assessments on Days 0 and 10 because caffeine may affect gastric emptying [43]. Hormonal contraceptives were also prohibited based on the potential for interference with acetaminophen metabolism [44].

### Treatments

Teduglutide 4 mg or placebo was administered by daily subcutaneous abdominal injection in a volume of 0.4 mL in the morning on Days 1 through 10. On Day 10, study drug was administered following an overnight fast and 3 hours before administration of a commercially available liquid meal providing 240 kcal and containing 10 g protein, 4 g fat, and 41 g carbohydrate (Boost®, Nestlé Healthcare Nutrition, Florham Park, NJ). To ensure treatment compliance, study medication was administered under the direct supervision of the site investigator.

Acetaminophen extra-strength liquid 1000 mg (30 mL) was administered on Days 0 and 10 immediately before a standard Boost meal and 3 hours after teduglutide or placebo on Day 10.

### Assessments

#### ***Pharmacodynamics of gastric emptying***

The primary study endpoint was a PK analysis of acetaminophen absorption in subjects receiving teduglutide or placebo. Blood levels of acetaminophen were measured at 0, 0.25, 0.5, 0.75, 1, 1.25, 1.5, 2, 3, 3.5, 4, 5, 6, 8, 10, 12, and 14 hours after acetaminophen administration on Days 0 and 10 (Table 1).

**Table 1:** Day 10 gastric emptying and pharmacokinetic schedule

**Example clock time**	**7:00**	**7:15**	**7:30**	**8:00**	**9:00**	**10:00**	**10:15**	**10:30**	**10:45**	**11:00**	**11:15**	**11:30**	**12:00**	**13:00**	**13:30**	**14:00**	**15:00**	**16:00**	**17:00**	**18:00**	**19:00**	**20:00**	**21:00**	**22:00**	**24:00**
Gastric emptying schedule, hours						0	0.25	0.50	0.75	1	1.25	1.5	2	3	3.5	4	5	6		8		10		12	14
Teduglutide administration	X																								
Acetaminophen administration						X																			
Standard meal^*^																X			X^*^						
Boost® meal						X																			

^*^Standard solid meal; dinner could have been given at any time following the last biomarker draw.

#### ***Safety***

Safety assessments included monitoring of adverse events (AEs), clinical laboratory tests, electrocardiogram, physical examination, and vital signs. For each AE recorded, an intensity level (ie, mild, moderate, or severe) was assigned. Mild AEs generally did not interfere with daily activities, required no special treatment, and were usually transient. Moderate AEs impaired daily activities but were usually easily resolved. Severe AEs interrupted daily activities and required vigorous therapeutic intervention. Serious AEs and AEs considered related to study drug were recorded through 30 days after the last study drug dose.

### Statistical analysis

The sample size of 36 was based on phase I considerations for hypothesis generation rather than power considerations. The intent-to-treat (ITT) population included all randomized subjects who received at least 1 dose of study medication and had at least 1 PK/pharmacodynamic (PD) measurement. The PK/PD population included all subjects from the respective population who had at least 3 PK measurements. The safety population included all randomized subjects who received at least 1 dose of study drug and who had any follow-up information.

For acetaminophen absorption, PK parameters estimated from plasma concentration (Days 0 and 10) included area under the plasma concentration versus time curve (AUC) from time 0 to the last measurable concentration (AUC_0–last_) and extrapolated to infinity (AUC_0–inf_), maximum plasma concentration (C_max_), and time to C_max_ (t_max_). PK parameters were estimated from the actual time of sampling and were calculated using noncompartmental analysis. Analysis of covariance (ANCOVA) using baseline (Day 0) values and sex as covariates was used to analyze the acetaminophen PK data and calculate differences in gastric emptying of liquids between teduglutide and placebo for AUC and C_max_ parameters. A rank-adjusted nonparametric ANCOVA was used for t_max_.

Safety data were summarized using descriptive statistics for quantitative data and frequency counts for qualitative data. AEs were coded using Medical Dictionary for Regulatory Activities terminology.

SAS/STAT® version 9.1 (SAS Institute, Cary, NC) was used to analyze the data.

## Results

### Subject disposition

Between September and November 2010, 36 subjects were randomized to receive teduglutide (n = 23) or placebo (n = 13). All subjects received at least 1 dose of study medication and had at least 1 PK measurement (ITT population). In the placebo group, 1 subject withdrew consent for personal reasons and discontinued from the study after 7 days of treatment. This subject was not included in the PK/PD population. Subjects were well matched for demographics and baseline characteristics between the 2 groups (Table 2).

**Table 2:** Subject demographics

**Demographic variable**	**Placebo (n = 13)**	**Teduglutide 4 mg (n = 23)**
Mean (SD) age, y	32.5 (7.0)	31.4 (7.4)
Mean (SD) weight, kg	80.9 (12.4)	70.3 (12.0)
Mean (SD) height, cm	173.6 (10.7)	169.0 (8.7)
Mean (SD) body mass index, kg/m^2^	26.7 (2.2)	24.5 (3.1)
Sex, n (%)		
Male	8 (62)	14 (61)
Female	5 (38)	9 (39)
Race, n (%)		
Black	4 (31)	5 (22)
White	9 (69)	18 (78)
Ethnicity, n (%)		
Hispanic or Latino	8 (62)	17 (74)
Not Hispanic or Latino	5 (38)	6 (26)

### Gastric emptying

**Figure 2:**
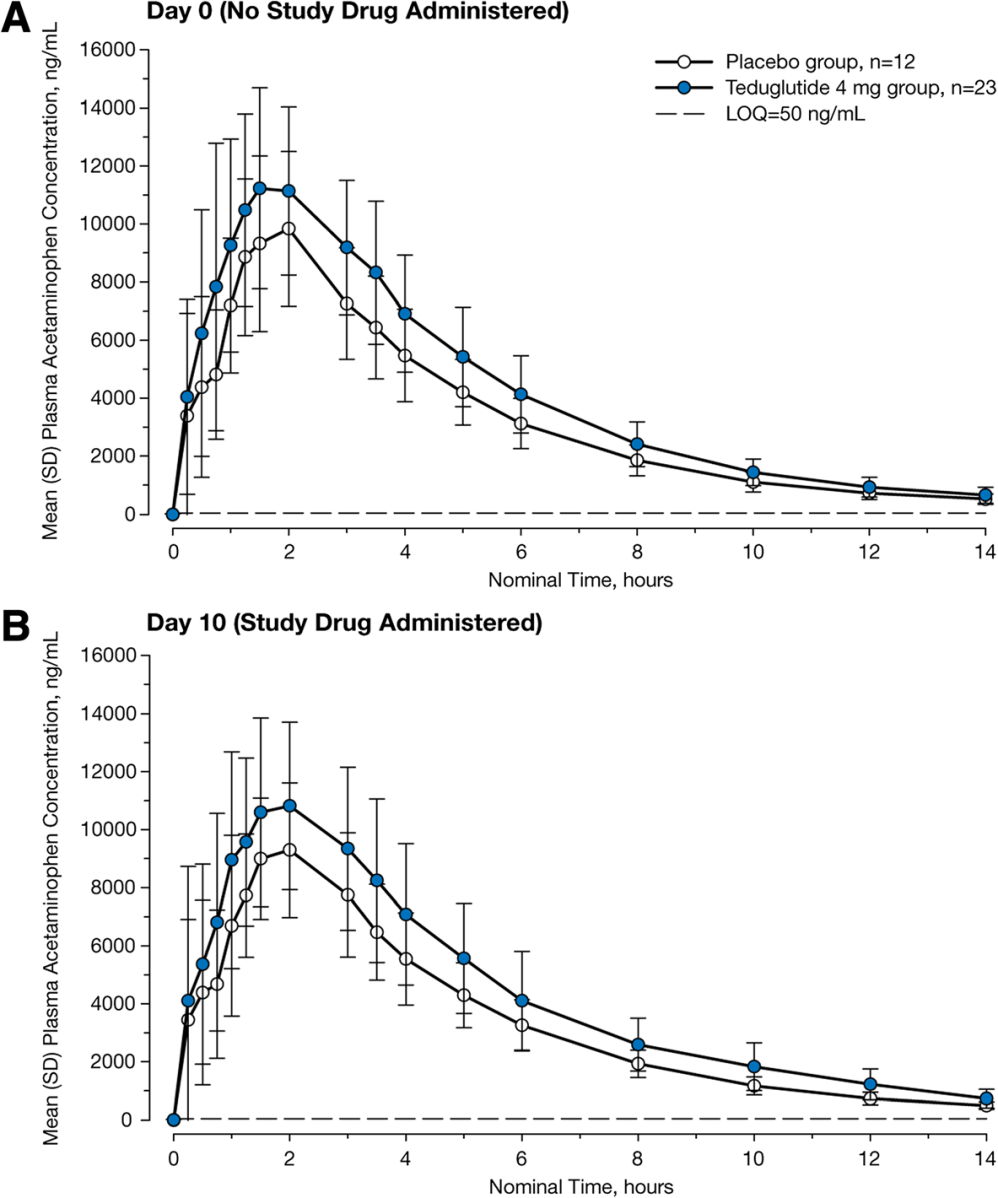
**Plasma acetaminophen concentrations vs time. A**, Day 0, before administration of study drug. **B**, Day 10, following 10 days of study drug administration. LOQ = limit of quantification.

Compared with placebo, teduglutide treatment for 10 days had no appreciable effect on gastric emptying of liquids as determined by acetaminophen PK parameters. Although acetaminophen absorption appears to be somewhat higher in the teduglutide group compared with the placebo group on both Day 0 and Day 10, the absorption curves for the teduglutide group and the placebo group on Day 10 were not significantly different from the corresponding curves on Day 0 (Figure 2A and B). The Day 10 absorption curves also did not differ significantly between the treatment groups. At Day 10, no significant differences were observed between the 2 groups in AUC_0–last_, AUC_0–inf_, C_max_, or t_max_ (Table 3). Within the teduglutide group, acetaminophen PK parameters were similar at Day 0 and Day 10 (AUC_0–last_, 60,105 ± 13,028 vs 60,984 ± 15,710 ng∙hour/mL, respectively; AUC_0–inf_, 63,693 ± 14,187 vs 65,279 ± 17,039 ng∙hour/mL; C_max_, 13,319 ± 3155 vs 12,677 ± 3821 ng/mL; t_max_, 1.6 vs 1.7 hour). Similarly, the placebo group showed no apparent differences in PK parameters at Day 0 versus Day 10 (AUC_0–last_, 47,551 ± 9483 vs 47,652 ± 9377 ng∙hour/mL, respectively; AUC_0–inf_, 50,360 ± 10,001 vs 50,036 ± 9706 ng∙hour/mL; C_max_, 11,124 ± 2939 vs 10,679 ± 2246 ng/mL; t_max_, 1.5 vs 1.5 hour).

**Table 3:** Acetaminophen pharmacokinetic parameters on day 10 (PK/PD population*)

**Pharmacokinetic parameter, mean ± SD**	**Placebo (n = 12)**	**Teduglutide 4 mg (n = 23)**	***P *** **value**
AUC_0–last,_ ng∙hour/mL	47,652 ± 9377	60,984 ± 15,710	0.32
AUC_0–inf,_ ng∙hour/mL	50,036 ± 9706	65,279 ± 17,039	0.26
C_max,_ ng/mL	10,679 ± 2246	12,677 ± 3821	0.28
t_max,_ hour	1.5	1.7	0.65

AUC = area under the curve; AUC_0–last_ = AUC from time 0 to the last measurable concentration; AUC_0–inf_ = AUC from time 0 extrapolated to infinity; C_max_ = maximum plasma concentration; PD = pharmacodynamic; PK = pharmacokinetic; t_max_ = time to C_max_.

*1 patient in the placebo group withdrew consent on Day 7 and therefore did not have PK/PD data for Day 10.

### Safety

Treatment-emergent AEs for subjects who received at least 1 dose of study drug with any follow-up information are shown in Table 4. Overall, 23 (64%) subjects experienced a treatment-emergent AE. There were no serious AEs, deaths, or discontinuations due to an AE during the study. Mild and moderate AEs were reported in 20 subjects (56%) and 3 subjects (8%), respectively; no severe AEs were reported. Changes from baseline in clinical laboratory parameters, electrocardiogram, physical examination, and vital signs were not clinically significant.

**Table 4:** Treatment-emergent adverse events reported in ≥5 % of subjects in the teduglutide group

**Variable, n (%)**	**Placebo (n = 13)**	**Teduglutide 4 mg (n = 23)**
Treatment-emergent AEs	9 (69)	14 (61)
Abdominal distention	2 (15)	8 (35)
Constipation	2 (15)	5 (22)
Headache	2 (15)	5 (22)
Abdominal pain	1 (8)	5 (22)
Nausea	0	5 (22)
Dyspepsia	0	2 (9)
Eructation	0	2 (9)
Extremity pain	0	2 (9)

AE = adverse event.

## Discussion

In this study, teduglutide administered at 4 mg/day for 10 days did not affect gastric emptying of liquids in healthy subjects as measured by acetaminophen PK. Previous studies evaluating gastric emptying following administration of GLP-2, a similar but distinct molecular entity, have yielded conflicting results [21,28-30]. Comparisons with and among these studies are hampered by heterogeneities in trial design, study populations, GLP-2 dosages, routes of GLP-2 administration, and timing of treatments. Nonetheless, results with teduglutide presented here are in agreement with 2 prior studies demonstrating no effect of infused native GLP-2 (0.75–2.25 pmol ∙ kg^–1^ ∙ min^–1^ for 180–390 minutes) on gastric emptying rate following a 250-to 310-kcal solid meal, as determined by either scintigraphic measurement or ^13^C-sodium octanoate breath test [21,28]. In contrast, 2 other studies have reported delayed gastric emptying with GLP-2 [29,30]. In one of these studies, conducted in healthy adults following a 7.5-kcal liquid meal, antral emptying time was increased by 9.5 minutes following GLP-2 (initial bolus of 4.5 pmol/kg followed by infusion at 1.0 pmol ∙ kg^–1^ ∙ min^–1^ for 60 minutes) compared with placebo (*P* = 0.049), as determined by ultrasound scanning. GLP-2 infused at a lower rate (0.5 pmol ∙ kg^–1^ ∙ min^–1^) after the initial bolus did not significantly affect antral emptying time [29]. Contrary to the modest results reported by Nagell et al., Jeppesen et al. described a 30-minute increase in time to 50% gastric emptying of solids following 35 days of treatment with subcutaneous GLP-2 (400 μg twice daily; *P* = 0.002) using a scintigraphic technique [30]. In this case, however, the study enrolled patients with SBS who may have had baseline disturbances in gastric emptying. For example, half of the patients in the Jeppesen study had an end-jejunostomy, which in a previous study was correlated with accelerated emptying of gastric liquids for patients with SBS [45]. Furthermore, end-jejunostomy patients have diminished intestinal secretion of GLP-2 [30,46]. For these reasons, GLP-2 may have a greater effect on gastric emptying in the end-jejunostomy population.

The liquid meal provided to study subjects contained fats in addition to carbohydrates and proteins. Previous studies suggest that the lipid phase empties more slowly than the aqueous phase in the context of a mixed solid-liquid meal [47,48]. However, when fats are homogenized with liquids before ingestion, as is the case with a liquid-only meal, both fats and the aqueous phase empty at the same rate [49]. Therefore, it is likely that the data from this study reflect simultaneous gastric emptying of both fats and the aqueous phase of the liquid meal.

Recently, rapid tachyphylaxis has been documented for GLP-1–dependent inhibition of gastric emptying in humans following prolonged infusion [50], prompting the question of whether the physiological effects of GLP-2 or teduglutide could also be affected by tachyphylaxis. Presently, however, no data are available concerning the potential for tachyphylaxis with GLP-2 or teduglutide with regard to gastric emptying. Of the 4 published studies evaluating the effects of GLP-2 on gastric emptying in humans, none provided multiple measurements of gastric emptying following GLP-2 administration [21,28-30]. The results presented in this study show no effect of teduglutide on gastric emptying following 10 days of subcutaneous administration; however, the study was not designed to evaluate gastric emptying rates earlier in the treatment period, or following multiple meals consumed within a period of less than 24 hours. However, several studies have demonstrated sustained biological activity of GLP-2 or teduglutide over extended treatment periods. For example, mice who received subcutaneous GLP-2 for 12 weeks showed a progressive increase in small bowel weight over the course of the study [17]. Patients with SBS treated with subcutaneous teduglutide (0.05 mg/kg/day) experienced continued reductions in PN/IV volume requirements throughout a 52-week study. Furthermore, 11 of 19 patients in the same study who were nonresponders following 24 weeks of teduglutide achieved a clinically significant response, defined as a ≥20% reduction in PN/IV volume from baseline, by Week 52 [51]. These data indirectly suggest continued improvement with long-term teduglutide treatment, with no dampening of response.

Teduglutide was well tolerated in this study. No unexpected safety signals were observed. AEs were generally mild, with no severe AEs reported. The most common AEs observed in this study were gastrointestinal related, which is consistent with the known mechanism of action of teduglutide. These results are also in agreement with clinical trials for teduglutide conducted in patients with SBS dependent on parenteral support. In two 6-month, placebo-controlled, phase III studies, gastrointestinal-related AEs were the most frequently reported class of AEs among patients receiving 0.05 mg/kg/day teduglutide [31,36].

This study was limited by the small sample size and use of healthy volunteers. Furthermore, the methods applied in this study assess gastric emptying of liquids only. However, the appearance of ingested acetaminophen in the plasma is an established and validated measure of gastric emptying kinetics [42] and has been used extensively in the analysis of GLP-1 receptor agonists [52-57].

## Conclusions

The results presented here suggest that teduglutide does not act to delay liquid-phase gastric emptying in healthy subjects. Therefore, teduglutide is unlikely to modulate the bioavailability of orally administered concomitant medications through inhibition of gastric motility in this population. However, the effects of teduglutide on gastric emptying in patients with SBS remain to be investigated.

## Abbreviations

AEs: Adverse events; ANCOVA: Analysis of covariance; AUC: Area under the plasma concentration versus time curve; AUC0–inf: Area under the plasma concentration versus time curve from time 0 extrapolated to infinity; AUC0–last: Area under the plasma concentration versus time curve from time 0 to the last measurable concentration; Cmax: Maximum concentration; CNS: Central nervous system; GI: Gastrointestinal; GLP: Glucagon-like peptide; ITT: Intent to treat; LOQ: Limit of quantification; PD: Pharmacodynamic; PK: Pharmacokinetics; PN/IV: Parenteral nutrition and/or intravenous fluid; SBS: Short bowel syndrome; SC: Subcutaneous; tmax: Time to maximum concentration.

## Competing interests

J K Berg was an employee of the PRACS Institute, which contracted with NPS to perform the study. E H Kim, B Li, B Joelsson, and N N Youssef are employees of NPS Pharmaceuticals, Inc. This study was funded in full by NPS Pharmaceuticals, Inc. Initial data analyses were undertaken by the PRACS Institute and supported by NPS Pharmaceuticals, Inc.

## Authors’ contributions

JB carried out the study. BL performed the statistical analyses. EK, BJ, and NY analyzed the data. All authors assisted in drafting and/or revising the manuscript and approved the final version of the manuscript.

## Pre-publication history

The pre-publication history for this paper can be accessed here:

http://www.biomedcentral.com/1471-230X/14/25/prepub
